# Myosins Are Differentially Expressed under Oxidative Stress in Chronic Streptozotocin-Induced Diabetic Rat Brains

**DOI:** 10.1155/2013/423931

**Published:** 2013-09-24

**Authors:** Luciana Karen Calábria, Alice Vieira da Costa, Renato José da Silva Oliveira, Simone Ramos Deconte, Rafael Nascimento, Washington João de Carvalho, Vanessa Neves de Oliveira, Carlos Alberto Arcaro Filho, Luciana Rezende Alves de Oliveira, Luiz Ricardo Goulart, Foued Salmen Espindola

**Affiliations:** ^1^Institute of Genetics and Biochemistry, Federal University of Uberlândia, Uberlândia, MG, Brazil; ^2^Federal University of Juiz de Fora, Governador Valadares, MG, Brazil; ^3^Federal University of Juiz de Fora, Juiz de Fora, MG, Brazil; ^4^Department of Chemistry, Physic, and Mathematic, University of Ribeirão Preto, Ribeirão Preto, SP, Brazil

## Abstract

Diabetes mellitus is a disease characterized by persistent hyperglycemia, which may lead to brain tissue damage due to oxidative stress and also contributes to neuronal death and changes in synaptic transmission. This study evaluated the effect of oxidative stress and the use of antioxidants supplementation on myosins expression levels in the brains of chronic diabetic rats induced by streptozotocin. Lipid peroxidation, antioxidant enzymes activities, and myosins-IIB and -Va expressions at transcriptional and translational levels were examined after 90 days induction. The chronic effect of the diabetes led to the upregulation of superoxide dismutase (SOD) and catalase (CAT) activities, and the downregulation of glutathione peroxidase (GPx), but there was no statistically significant increase in the malondialdehyde (MDA) levels. These alterations were accompanied by high myosin-IIB and low myosin-Va expressions. Although the antioxidant supplementation did not interfere on MDA levels, the oxidative stress caused by chronic hyperglycemia was reduced by increasing SOD and restoring CAT and GPx activities. Interestingly, after supplementation, diabetic rats recovered only myosin-Va protein levels, without interfering on myosins mRNA levels expressed in diabetic rat brains. Our results suggest that antioxidant supplementation reduces oxidative stress and also regulates the myosins protein expression, which should be beneficial to individuals with diabetes/chronic hyperglycemia.

## 1. Introduction

Diabetes mellitus is a multifactorial disease characterized by chronic hyperglycemia resulting from abnormalities in insulin action and/or insulin secretion [1]. Research evidences support that both acute and chronic hyperglycemia produce negative impacts on the central nervous system leading to tissues damage [2, 3]. One mechanism behind this neuronal injury is oxidative stress, due to the excessive free radical generation from the oxidation of elevated intracellular glucose levels [4].

The brain contains large amounts of enzymes to protect against oxidative damage [5]. Endogenous antioxidant system, including enzymatic (glutathione peroxidase, superoxide dismutase, and catalase) and nonenzymatic (vitamin E, vitamin C, glutathione, and uric acid) antioxidants, offers protection to cells and tissues against glucose-induced oxidative injury in diabetics [6–10].

The enhancement on oxygen free radical in brain during hyperglycemia [11] contributes to increased neuronal death trough protein oxidation, DNA damage, and peroxidation of membrane lipids [12] as well as changes in synaptic transmission. These alterations could lead to abnormal synaptic plasticity and cognitive impairments observed in experimental diabetes [13, 14].

Myosins have been reported to be sensitive to oxidative damage [15, 16]. These proteins constitute a family of molecular motors that contains many classes and isoforms, which differ in their cellular distribution and function [17, 18]. Among the myosin classes identified so far, the family classes II and V have been best characterized in neurons and are implicated in a wide variety of cellular functions in the nervous system, including neuronal migration, growth cone motility, neuronal morphogenesis, axonal transport, synaptic and sensory functions, Glut4 vesicle trafficking, and in acute diabetes mellitus [19–25].

The rationale for using certain vitamins and minerals, or natural antioxidants, in the prevention and management of diabetes, is largely based on animal experiments and epidemiologic studies [26–28]. Diabetes is associated with increased oxidative stress, and this fact, raises the interest of using antioxidant supplements in individuals with diabetes in an attempt to prevent long-term complications [27].

In spite of the recent studies that have shown relationship between diabetes and the expression of molecular motors, as myosin-IIB [21], myosin heavy-chain [29], myosin-Va [23, 25], and myosin-IXB [30], the pathways linked among myosin expression, oxidative stress, and diabetes mellitus are still unclear. The present study aimed to evaluate the effect of the use of antioxidant supplementation on myosins expression in brain of chronic streptozotocin-induced diabetes experimental rat model.

## 2. Materials and Methods

### 2.1. Animals

All experimental procedures were conducted in accordance with the ethical principles of the Brazilian Academy of Animal Experimentation and approved by the Committee of Ethics in Animal Experimentation from the University of Ribeirão Preto, UNAERP (066/09). Twenty-four male Wistar rats (*Rattus norvegicus*) (weight: 210–260 g) were housed under standard conditions (22 ± 1°C, humidity 60 ± 5%, 12 h light/12 h dark cycle) with food and water ad libitum on the Central Biotery of UNAERP.

### 2.2. Induction of Diabetes Mellitus

After one week of acclimatization, the rats were subjected to a 24-h starvation. The animals were then anesthetized by intraperitoneal injection of xylazine/ketamine (1 : 1 v/v), and then streptozotocin (40 mg/kg body weight), freshly dissolved in 0.01 M citrate buffer, pH 4.5, was injected into the penile vein (2 mL/kg). Food was denied for 90 min after injection. Ten days after the streptozotocin or buffer injection, blood glucose was determined, and animals with blood glucose above 200 mg/dL were scored diabetic. Animal (*n* = 24) weight was monitored daily until the decapitation and surgical removal of brains 90 days after diabetes induction and/or supplementation.

### 2.3. Group Distribution and Supplementation of the Rats

The rats were distributed randomly in three groups (*n* = 8, each): nondiabetic (ND), diabetic (D), and diabetic supplemented (SD). All animals were fed diets based on a modified AIN93G rodent diet, except the supplemented diabetic group that was fed with additional calcium (2.5-fold), zinc (500 mg), and vitamin E (20-fold), following the principles of the American Institute of Nutrition [31].

### 2.4. Sample Collection and Tissue Preparation

The brains of all animals were quickly removed and washed with chilled normal saline and immersed in liquid nitrogen. Simultaneously, the blood was also collected from the portal vein to confirm the glucose levels. For oxidative stress markers and western blotting analyses, each of the brains was individually homogenized on ice in homogenization buffer (50 mM Tris-HCl, pH 7.5, 2 mM dithiothreitol, 1 mM benzamidine, 0.5 mM phenylmethanesulfonyl-fluoride, 0.1 M aprotinin, 20 *μ*g/mL leupeptin, 0.1 mM pefabloc). The homogenates were centrifuged at 15,000 ×g for 30 min at 4°C, and total protein concentration in the supernatant samples was measured following the Bradford assay [32].

### 2.5. Oxidative Stress Markers Analysis

Glutathione peroxidase (GPx) and superoxide dismutase (SOD) activities were measured using kit. *CAT activity*, Catalase (CAT), activity was assessed spectrophotometrically monitoring hydrogen peroxide decomposition at 240 nm [33], and the substrate concentration was 20 mM for brain measurements. Lipid peroxidation in tissue was determined by measuring the presence of malondialdehyde (MDA) using the thiobarbituric acid test (TBARS).

### 2.6. Western Blotting

Aliquots of supernatant samples were solubilized in a small volume of electrophoresis sample buffer containing an additional 100 mM Tris-HCl, pH 8.0, and 25% glycerol. Supernatant samples containing 20 *μ*g of protein were analyzed by SDS-PAGE with a 12% acrylamide-bisacrylamide, and gels were electroblotted on nitrocellulose membranes in Tris-glycine buffer [34]. Blots were incubated with 5% dried milk in PBS-T (50 mM Tris-HCl, pH 8.0, 150 mM NaCl, 0.05% Tween 20) then probed with antimyosin-IIB and antimyosin-Va primary antibodies diluted to 0.2 *μ*g/mL. The polyclonal antibodies were generated in rabbits against nonmuscle myosin II purified and myosin-V head domain from chicken brain and purified by affinity to antigen immobilized on nitrocellulose strips as described previously [35–37]. *β*-actin antibody was used as control. Blots were incubated with a peroxidase-conjugated antirabbit IgG (diluted 1 : 2000). Antibodies bound to the membranes were visualized by chemiluminescence. The intensity of the protein bands was analyzed and results were expressed as percentage of total content.

### 2.7. mRNA Expression Levels Using qRT-PCR

Total RNA was isolated from nondiabetic, diabetic, and supplemented diabetic brains separately and then resuspended in DEPC-treated water. Its quality and quantity were established by reading the optical density of each sample at 260 and 280 nm and agarose gel electrophoresis. One microgram of total RNA was reverse transcribed at 37°C for 1 h in a 20 *μ*L reaction mixture containing the first strand buffer, 40 U Murine Monoley Leukemia Virus Reverse Transcriptase, 0.25 mM of each dNTP, 10 U RNAsin, 0.5 mM DTT, and 126 pmol hexamer random primers. Standards curves were prepared by cloning PCR products of *MYH10*, *MYO5A*, and the housekeeping beta-2-microglobulin (*B2M*) fragments using TOPO TA Cloning Dual Promoter Kit. The recombinant plasmid DNA was isolated and sequenced using automatic sequencer. The sequencing reaction was carried out using the DyEnamic ET Dye Terminator Cycle Sequencing Kit. The concentration of plasmid was measured and the copy numbers were calculated [38]. Serial dilutions of each of standard curves were made in the range of 10 to 10^7^ copies per *μ*L for *MYH10*, *MYO5A*, and *B2M*. The reaction efficiency was calculated according to the formula *E* = (10^−1/slope^ − 1) × 100, where the log of the each dilution was plotted with Δ*C*_*T*_ of housekeeping and target genes, *R* ^2^ ≥ 0.99 and slope value about 3.32 were admitted to reaction efficiency of 100%. The qPCR assay was performed by using the realtime PCR system and SYBR Green qPCR Master Mix reagent. Primers were designed between exons junctions to avoid amplification of contaminating the genomic DNA. For *B2M* fragment amplification, the set of primers were: 5′-CGT CGT GCT TGC CAT TCA-3′ and 5′-TCC TCA ACT GCT ACG TGT CTC AG-3′. The *MYH10* forward and reverse primers were respectively: 5′-CCA TGC CGG AGA ACA CAG T-3′ and 5′-AAG CCC AGA CCA AAG AGC AG-3′. The *MYO5A* forward and reverse primers were respectively: 5′-ATT GAG GCT CGC TCT GTG GA-3′ and 5′-ACG CAA AGT GGA TGA GCA GA-3′. The relative expression of each specific product was calculated by 2^−ΔΔ*C*_*T*_^ (*C*_*T*_ = fluorescence threshold value; Δ*C*_*T*_ = *C*_*T*_ of the target gene − *C*_*T*_ of the reference gene (*B2M*); ΔΔ*C*_*T*_ = Δ*C*_*T*_ of the target sample − Δ*C*_*T*_ of the calibrator sample). All samples were run in duplicates. For expression analysis of the *MYO5A* gene, the cDNA was 4-fold diluted.

### 2.8. Statistical Analysis

All values obtained are expressed as mean ± SEM. Data were initially analyzed by one-way analysis of variance (ANOVA). When differences were detected by ANOVA, these sets of nondiabetic, diabetic, and supplemented diabetic rats were compared using Student's *t*-test or Tukey's test to determine the statistical significance, which was assumed to be different when the comparison showed a significance level of *P* < 0.05.

## 3. Results

### 3.1. Blood Glucose Levels and Body Weight

Blood glucose and body weight (*n* = 8 rats/subgroup) were measured (Table 1). Streptozotocin injection produced diabetic rats with consistent high levels of blood glucose. The diabetic and supplemented diabetic rats had significantly higher blood glucose levels (*P* < 0.001) and lower body weight (*P* < 0.05) than nondiabetic rats. When time was compared, before and after treatment, diabetic rats showed a decreased body weight after 90 days; however, the nondiabetic and supplemented diabetic rats showed an increased body weight (*P* < 0.001).

### 3.2. Antioxidant Defense System Enzymes and Lipid Peroxidation

**Figure 1:**
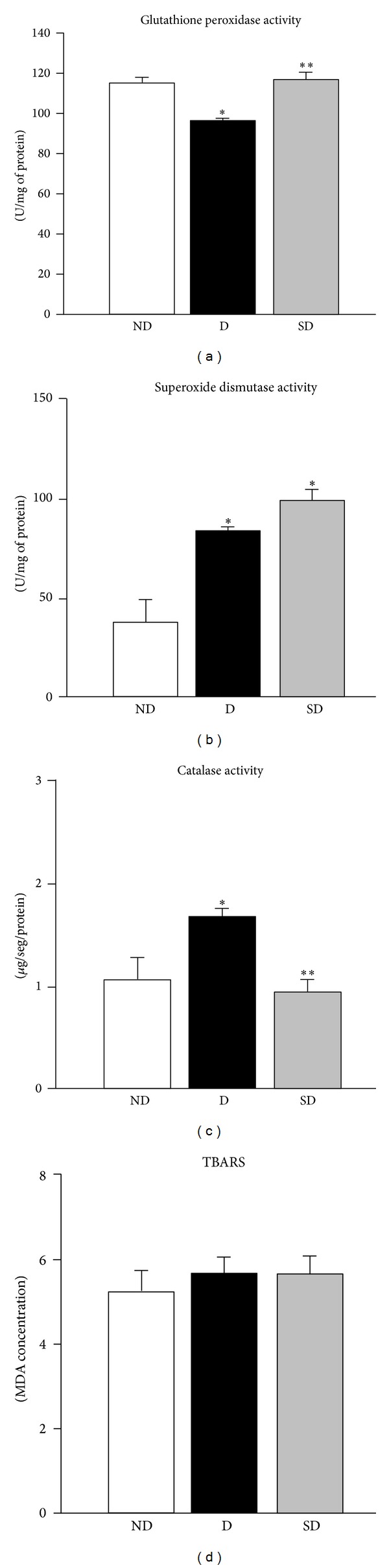
Supplementation effects on antioxidant enzymes activity and lipid peroxidation in diabetic rat brain. Data are mean ± SEM, *n* = 4 rats/subgroup. Significant differences: *compared to nondiabetic (ND) (GPx and SOD- *P* < 0.001; CAT- *P* < 0.05); **diabetic (D) *versus* supplemented diabetic (SD) (GPx- *P* < 0.001; CAT- *P* < 0.05).

Glutathione peroxidase (GPx), superoxide dismutase (SOD), and catalase (CAT) activities in brain of nondiabetic, diabetic and supplemented diabetic rats were presented in Figure 1 (*n* = 4 rats/subgroup). Streptozotocin caused a noticeable reduce of cerebral GPx levels in diabetic rats at 90-day post induction (*P* < 0.001). Cerebral SOD activity was higher in diabetic, and supplemented diabetic rats than nondiabetic (*P* < 0.001). Cerebral CAT activity was significantly elevated in diabetic rats when compared to the nondiabetic (*P* < 0.05). Besides, the increase in CAT activity in diabetic rats was accompanied by a significant decrease in the GPx activity in the brain. Moreover, supplementation protected against hyperglycemic-induced increase in GPx (*P* < 0.001) and CAT (*P* < 0.05) activities by maintaining the enzyme levels in the diabetic rat brains, similar to nondiabetic. Otherwise, the levels of malondialdehyde (MDA) were markedly elevated in nondiabetic and diabetic rats. No difference was observed between rats at the 90-day period, and supplementation seems to be inefficient and does not affect the lipid peroxidation status in the brain.

### 3.3. Determination of Changes in Myosins Protein Expression Levels

**Figure 2:**
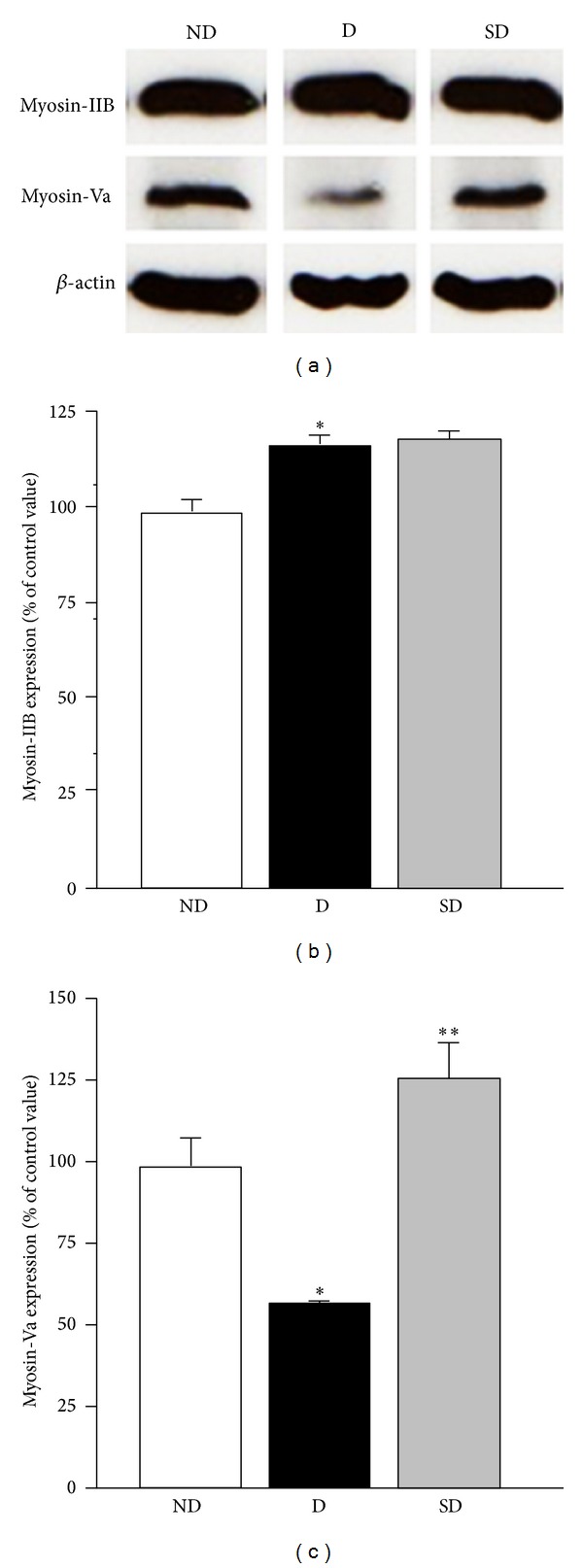
Supplementation and diabetes effects on myosin-IIB and myosin-Va protein levels in rat brains. The amount of myosin proteins showed on the immunoblot was determined densitometrically and expressed as a related percentage of the groups (*n* = 4 rats/subgroup). Values represent mean ± SEM. Significant differences: *compared to nondiabetic (ND) (Myosin-IIB- *P* < 0.05; Myosin-Va- *P* < 0.005); **diabetic (D) *versus* supplemented diabetic (SD) (Myosin-Va- *P* < 0.005).

In order to check if the myosin proteins levels could be altered after 90 days of diabetes or supplementation duration, the amount of myosins IIB (210 kDa) and Va (190 kDa) were estimated by Western blotting (*n* = 4 rats/subgroup).  Figure 2 displays the combined results of immunoblots and densitometrically quantitated myosins immunoreactivity represented as percentage of nondiabetic from diabetic and supplemented diabetic rats. In a general manner, diabetes increased the myosin-IIB protein level in the brain (*P* < 0.05) with 18% higher than nondiabetic values. Myosin-Va protein level decreased by 45% in diabetic rat brains as compared to nondiabetics in 90-day period (*P* < 0.005). No significant differences were observed for myosin-IIB protein level in supplemented diabetic, but the antioxidant supplementation restored the myosin-Va protein content in the diabetic brain and increased by 25% as compared to nondiabetics (*P* < 0.005).

### 3.4. Determination of Changes in Myosins mRNA Expression

**Figure 3:**
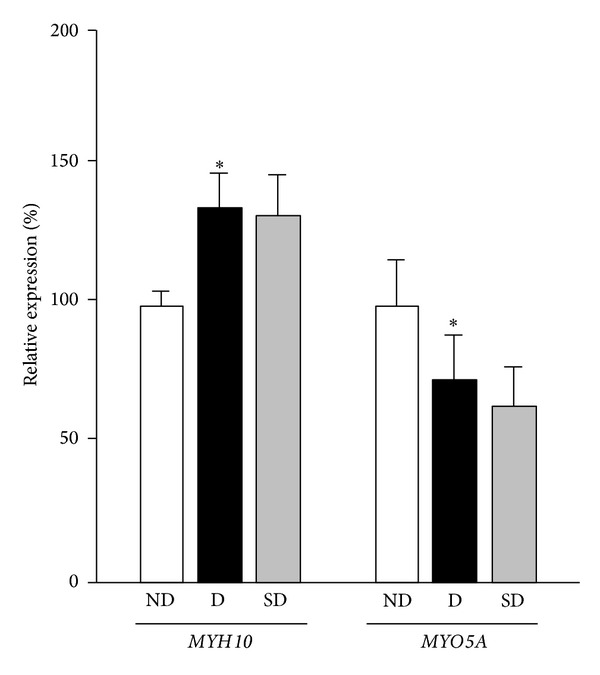
Diabetes and supplementation effects on *MYH10* and *MYO5A* mRNAs expression in rat brains. Relative expression of *MYH10* and *MYO5A* mRNAs in brain samples of diabetic (D) and supplemented diabetic (SD) compared to nondiabetic (ND) rats. (*) *P* < 0.05. Values represent mean ± SEM (*n* = 4 rats/subgroup).

Real time PCR (*n* = 4 rats/subgroup) was performed to measure changes in myosins gene expression for both *MYH10* (myosin-IIB) and *MYO5A* (myosin-Va) at 90-day period (Figure 3). Changes in gene expression were calculated based on the 2^−ΔΔ*C*_*T*_^ method with beta-2-microglobulin(*B2M*) as an endogenous control. Diabetic showed increased *MYH10 *mRNA expression as compared to nondiabetic (25%, *P* < 0.05). *MYO5A* mRNA was decreased in diabetic as compared to nondiabetic (30%, *P* < 0.05). However, no significant differences in *MYH10* and *MYO5A* mRNA expression were observed between diabetic and supplemented diabetic in both periods. Amplification efficiency was tested by standard curves for *Rattus novergicus B2M* (*R* ^2^ = 0.9997), *MYH10* (*R* ^2^ = 0.996), and *MYO5A* (*R* ^2^ = 0.9999) generated by plotting the value of *C*_*T*_ cycle *versus* the log of plasmid concentration (from 10^3^ to 10^6^ copies).

## 4. Discussion

Streptozotocin-induced diabetes is a well-documented model of experimental diabetes in rats. It provides a relevant example of endogenous chronic oxidative stress as a result of hyperglycemia [39]. In the present study, streptozotocin treatment produced a significant increase in blood glucose levels along with a reduction in body weight. These results are in accordance with other studies, which showed that diabetes mellitus increase plasma glucose levels and decrease body weight of diabetic rats [40–44]. In fact, supplementation did not reduce blood glucose levels and this may have contributed in part to the nonsuppressive effect of antioxidants on oxidative stress, though the body weight had been reduced in diabetic rats.

We have also analyzed hyperglycemia-induced oxidative stress in rat brain. The increase in free radical generation along with the depletion of antioxidants is the mechanism involved in diabetes-induced oxidative stress. There are evidences of alterations in free radical metabolism [45] and in the antioxidant parameters status [46, 47] during diabetes in various tissues. Moreover, there are contradictory results in the literature regarding the effect of hyperglycemia-induced diabetes on antioxidant enzymes activities [48–50]. Thus, the current study show the effects of the concomitant use of vitamin E, calcium, and zinc as antioxidants on the activities of defense enzymes, such as glutathione peroxidase (GPx), superoxide dismutase (SOD), and catalase (CAT) in the rat brains after 90 days of supplementation.

GPx and SOD are the first line of defense against free radical attacks. Their function is to catalyse the conversion of superoxide radicals to hydrogen peroxide [51]. Cerebral levels of GPx, a potent endogenous antioxidant, were reduced in diabetics at 90-day after induction. This result was also shown in diabetics at 21-day after induction [25]. However, the activity of GPx that has been shown increased in brain [42, 52, 53]. This increase was also observed after supplementation. GPx is responsible for the decomposition of hydrogen peroxide and other lipid peroxides, and it is possible that supplementations have avoided the GPx activity reduction in induced hyperglycemia, maintaining the enzyme levels in brain of diabetics, similar to nondiabetic rats.

The increase in cerebral SOD activity was observed in diabetic rats at 90-day period, which can lead to an important elimination of superoxide ions that inhibit the formation of hydroxyl radical in tissues. The increase SOD activity in type 2 diabetic mice brain has been reported [54, 55] to be a putative protection mechanism of oxidative stress. In the meantime, the SOD activity also appears to be decreased in diabetic rats [42, 56, 57].

GPx activity in the supplemented diabetic rats remained at same levels as in nondiabetic rats, whereas the SOD activity increased significantly compared to nondiabetic. The alteration of antioxidant enzymes GPx and SOD levels in the diabetic rats could be attributed to peroxidative damage of the tissues caused by streptozotocin-induced hyperglycemia [58], while supplementation with antioxidants contributed to maintaining the optimum condition of enzyme activity in the cellular organelles, by protecting them from peroxidation in chronic diabetes.

Besides, the SOD and CAT are also the major antioxidant enzymes against oxidative stress and appear to be decreased in diabetic rats [57, 59]. CAT is responsible for the catalytic decomposition of hydrogen peroxide formed in cellular metabolism in oxygen and water molecules. Its decreased activity at the chronic stage (90 days) might indicate a fine modulation of the CAT activity in order to protect the brain against free radicals and may also advocate for the importance of antioxidant supplementation at this stage for a better tissue response and protection. Although there are discrepancies in the levels of antioxidant enzymes reported in diabetic rats [60, 61], our results have shown an important balance between GPx and CAT activities.

These antioxidant enzymes have a complementary catalytic activity leading to a reduced MDA concentration, which represents lipid peroxidation products in tissue and blood. In the present study, we did not observe that MDA levels increased in diabetic rats at 90-day period, although this effect is to be observed in diabetic rats at 21-day after induction [25]. Nevertheless, supplementation was not sufficient to reverse those elevated levels of lipid peroxidation. It is well known that hyperglycemia increases lipid peroxidation, which may contribute to long-term tissue damage [62]. Besides, brain seems to be more sensitive than the other tissues, and hence the increase in GPx activity after 90 days supplementation was not sufficient to reduce MDA concentrations and thus to protect this tissue from lipid peroxidation.

Minerals (calcium and zinc) and vitamin E were added in diet of the diabetic rats to act as an antioxidant supplement in animals. Evidence from clinical studies has suggested that calcium could indirectly affect glucose metabolism, which would be desirable for diabetes prevention [63, 64]. Similarly, zinc acts as an antioxidant in order to reduce oxidative stress, it is essential for the function of SOD [65] and is also involved in insulin synthesis [66] which are altered in diabetes [67, 68]. Treatment with zinc significantly reduces astrocytosis [69], elevates SOD activity [70], and may be able to prevent diabetes effects in brain or against various damaging effects. Additionally, vitamin E possesses antioxidant activity [1, 71, 72], and neuroprotective action [73], it plays a role in hyperglycemia prevention [74, 75] and reduces lipid peroxidation in the brain [11]. In contrast, there are evidences that excessive dietary zinc and vitamin E intake can induce pathological conditions associated with oxidative stress [76, 77]. In regards to diabetes prevention through supplementation with micronutrients, the current evidences do not allow any particular recommendation for mineral or vitamin supplementation on a large scale. Given that diabetes is a condition of increased oxidative stress, antioxidant therapy may represent a potential coadjuvant to antidiabetic pharmacological treatment by improving the glucose metabolism [27]. 

On the other hand, our results have evidenced that chronic effect of diabetes in brain led to an upregulation of GPx activity, and a downregulation of SOD and CAT activities, as well as a higher lipid peroxidation levels in nondiabetic rats after 90 days. Our findings are consistent with previous reports [78–81], although another study has revealed that GPx and CAT activities are relatively unaffected by age [79]. Moreover, the supplementation seems to balance the antioxidant enzymes though no alter lipid peroxidation levels.

Diabetes affect synaptic plasticity and neurotransmission in rats, and these results are well recognized such as neurophysiological and structural changes associated mainly with cognitive deficits and Alzheimer disease [14]. Degenerative changes of neurons and glia have been reported and suggested that alteration in synaptic transmission could contribute to cognitive impairments observed in diabetics [13, 43, 80]. Besides, oxidative stress induced by hydrogen peroxide induces cytoskeletal reorganization and significantly enhances the association of myosin to actin filaments [81]. Myosin has also been reported to be a particularly sensitive target of oxidative damage [15, 16, 52, 82], although a direct link between oxidative stress and myosin dysfunction has yet to be established.

Multiple lines of evidence suggest that myosins may be involved in the regulation of synaptic vesicles. Myosin-IIB modulates neurotransmitter release from synapses [83] while myosin-Va mediates synaptic vesicle trafficking [84]. We recently demonstrated that acute diabetes (20 days) alters protein and mRNA expression of myosin-IIB and myosin-Va in the rat brains [21, 23]. In the present study, we revealed an increased protein and mRNA expression of myosin-IIB and a decreased one of myosin-Va in chronic diabetic rat brains (90 days). It is possible that these translational and transcriptional changes are due to low insulin level and high glucose level in circulation providing a possible dysfunction in vesicle/organelle movement [21, 23].

Diabetic rats restored protein myosin-Va levels after supplementation when compared to control (nondiabetic rats), and no differences were found on myosin-IIB. This indicates that chronic hyperglycemia induces effects in the protein expression of myosins on brain, and the antioxidant balance (endogenous and exogenous) and reactive oxygen species improves myosin-Va protein level. Although supplementation did not restore the mRNA expression of myosin-Va, it is possible that it has promoted an increase on the recruitment of this molecular motor but not an overexpression.

## 5. Conclusion

In conclusion, this study contributes to yielding some insights across the association and alterations in myosins expression and antioxidant enzymes in chronic diabetic rat brains, and it suggests that antioxidant supplementation reduces oxidative stress and also regulates the myosins expression levels, which should be beneficial to individuals with diabetes/chronic hyperglycemia. Considering the brain tissue is heterogenous and it is composed of different cell types with diverse functions, further molecular investigations are needed to elucidate the regulation pathways of antioxidant enzymes through myosins expression in different brain regions during diabetes.

## Figures and Tables

**Table 1:** Effect of diabetes and supplementation on blood glucose and body weight after 90 days (*n* = 8 rats/subgroup).

Parameters	90 Days		
	Nondiabetic	Diabetic	Supplemented diabetic
Blood glucose (mg/dL)			
Initial	89.25 ± 2.28	398.88 ± 19.09^a^	552.38 ± 2.38^a^
Final	98.38 ± 1.86	362.25 ± 38.45^a^	467.13 ± 39.39^a^
Body weight (g)			
Initial	257.50 ± 3.32	249.88 ± 4.38	221.75 ± 4.48
Final	362.38 ± 18.55^c^	222.63 ± 25.69^b^	253.75 ± 31.15^b^

Data are mean ± SEM.

^a^ *P* < 0.001, significantly different from nondiabetic.

^b^ *P* < 0.05, significantly different from nondiabetic.

^c^ *P* < 0.001 initial versus final.
